# Low Expression of TBX4 Predicts Poor Prognosis in Patients with Stage II Pancreatic Ductal Adenocarcinoma

**DOI:** 10.3390/ijms12084953

**Published:** 2011-08-03

**Authors:** Meijuan Zong, Meng Meng, Liang Li

**Affiliations:** 1 Zibo Vocational Institute, Xishou Liantong, Zibo 255314, China; 2 School of Resources & Environment Engineering, Shandong University of Technology, Zhangdian Zhangzhou 12, Zibo 255049, China; E-Mail: drmmeng@126.com; 3 Zibo Central Hospital, Zhangdian Gong Qingtuan 54, Zibo 255036, China; E-Mail: drliangli@163.com

**Keywords:** pancreatic cancer, TBX4, methylation, prognosis, survival

## Abstract

This study was designed to investigate the expression of the T-box transcription factor 4 (TBX4), a tumor biomarker that was previously identified by proteomics, in pancreatic ductal adenocarcinoma (PDAC) and evaluate its clinical utility as a potential prognostic biomarkers for PDAC. The expression of TBX4 was detected in 77 stage II PDAC tumors by immunohistochemistry, and the results were analyzed with regard to clinicopathological characteristics and overall survival. Moreover, Tbx4 promoter methylation status in primary PDAC tumors and normal adjacent pancreas tissues was measured by bisulfite sequencing. Among 77 stage II PDAC tumors, 48 cases (62.3%) expressed TBX4 at a high level. No significant correlation between TBX4 expression and other clinicopathological parameters, except tumor grade and liver metastasis recurrence, was found. The survival of patients with TBX4-high expression was significantly longer than those with TBX4-low expression (P = 0.010). In multivariate analysis, low TBX4 expression was an independent prognostic factor for overall survival in patients with stage II PDAC. TBX4 promoter methylation status was frequently observed in both PDAC and normal adjacent pancreas. We conclude that a low level of TBX4 expression suggests a worse prognosis for patients with stage II PDAC. Down-regulation of the TBX4 gene in pancreas is less likely to be regulated by DNA methylation.

## 1. Introduction

Pancreatic ductal adenocarcinoma (PDAC) represents one of the most aggressive malignancies with a high mortality rate. It is the fourth leading cause of cancer death in the United States, and the sixth leading cause of death from malignant disease in China [1,2]. PDAC is not sensitive to radiation therapy and chemotherapy, and surgical resection remains its only curative option. However, because of the lack of typical symptoms and/or physical signs indicating early diagnosis, only 20% of patients diagnosed with PDAC are candidates for surgical resection. Despite curative resection, the long-term survival rate of these PDAC patients is still low (less than 20%), with a median overall survival of 18 to 24 months [3,4]. Some clinicopathological parameters such as tumor size, stage, and lymph node metastasis have been proven to be reproducible prognostic determinants in pancreatic cancer. However, all these factors are far less than sufficient to explain the individual variability, therefore, there is still a need for reliable prognostic markers for the patients with PDAC who undergo resection.

T-box transcription factor 4 (TBX4) was first described as a transcription factor involved in the regulation of hindlimb outgrowth and specification during embryonic development [5,6]. In a previous study, we identified TBX4 up-regulated in PDAC tissues by a proteomic approach [7]. It was the first time that TBX4 was associated with cancer. Kelemen *et al*. [8] recently reported that a TBX4 polymorphism may contribute to breast cancer risk. TBX4 is located on chromosome 17q23, which has been shown to be amplified in breast cancer and pancreatic cancer. TBX4 is very similar to TBX5 in its genomic organization and function during limb development. Most recently, Yu *et al*. [9] found that TBX5 may exert its role as a functional tumor suppressor by inducing tumor cell apoptosis and suppressing tumor cell proliferation and metastasis. Furthermore, loss of TBX5 expression in colon cancer is epigenetically controlled by promoter methylation and associated with poor prognosis [9]. Based on the above evidence, we speculate that TBX4 might also play a putative role in the development of pancreatic cancer. However, the clinicopathological and prognostic significance of TBX4 for PDAC patients have not been well characterized; most resectable pancreatic cancer are in stage II, and there is very little information on the prognosis of this subgroup of PDAC patients. Therefore, in this study, we examined TBX4 expression in 77 stage II PDAC tumors to determine whether TBX4 expression was associated with survival and other clinicopathological features in this resectable subgroup. Moreover, the promoter methylation status in PDAC tumors and normal adjacent pancreas tissues was also examined to determine whether TBX4 expression in pancreatic cancer was epigenetically controlled.

## 2. Results and Discussion

### 2.1. Expression of TBX4 in PDAC Tissues and Normal Adjacent Pancreas

**Figure 1.**
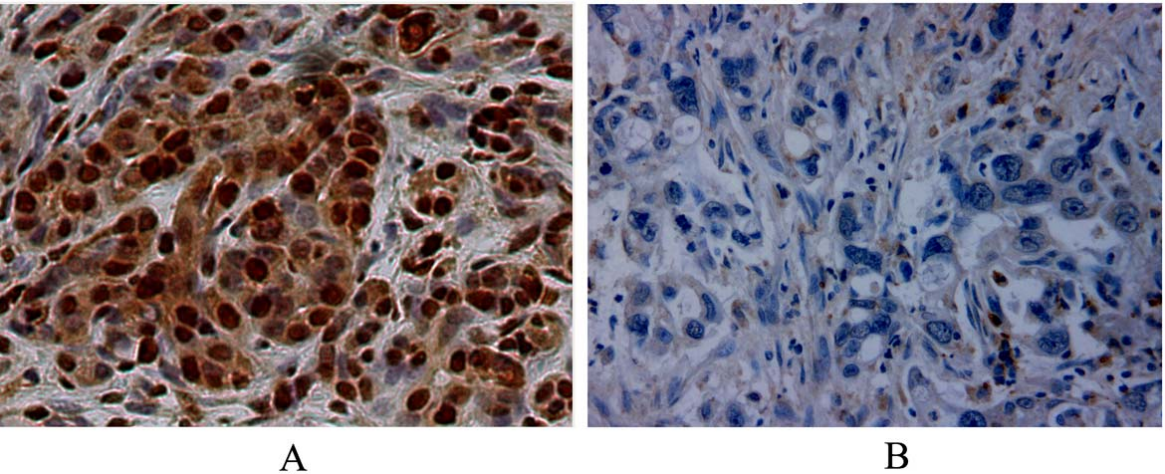
Representative micrographs showing pancreatic ductal adenocarcinoma (PDAC) with high TBX4 expression with cytoplasmic and nuclear staining (**A**) and low TBX4 expression (**B**). Original magnification, ×400.

Immunohistochemical stain for TBX4 showed cytoplasm and nuclear staining patterns in both malignant and normal pancreatic ductal epithelial cells, but not in acinar cells, islet cells or stromal fibroblasts. Fifteen PDAC tumors showed no TBX4 staining while the other 62 PDAC cases showed TBX4 expression with different staining density. The mean percentage of TBX4 labeling cancer cells to total cancer cells (labeling ratio) in 77 PDAC samples was 38.8% ± 16.4%. None of the normal pancreatic ducts showed a TBX4 labeling ratio of more than 25%. In this study, 48 PDAC cases with a labeling ratio ≥25% were classified as high level of TBX4 expression, wherease those showing a labeling ratio <25% were classified as low level of TBX4 expression. Representative micrographs of PDAC tumors with high and low level of TBX4 expression are shown in Figure 1.

### 2.2. Correlation of Clinicopathological Features with TBX4 Expression

Table 1 shows the relationship between TBX4 expression level and other clinicopathological factors of the study population. TBX4 expression levels showed an inverse correlation with higher grading (P = 0.015). Better differentiated PDAC tumors (Grade 1 and 2) more frequently expressed high level of TBX4 expression (45/48, 93.8%) than their poorly differentiated (Grade 3) counterparts (3/48, 6.2%). Liver metastasis recurrence was more frequently seen in the TBX4-low expressing group than the TBX4-high group (51.7% *vs*. 20.8%, P = 0.005). No significant association was identified between TBX4 expression and patient’s age, gender, tumor size, margin status, lymphnode metastasis and angiolymphatic invasion.

### 2.3. Correlation of TBX4 Expression Level and Overall Survival

**Figure 2.**
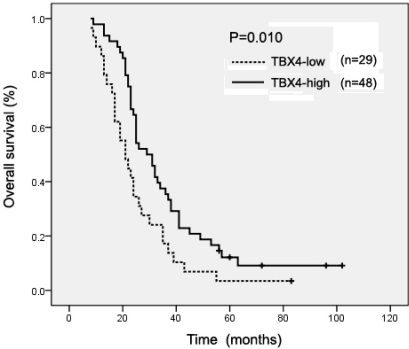
Kaplan–Meier survival curves show that TBX4-low expression PDAC patients had a shorter survival than those with TBX4-high expression. The difference is statistically significant based on the log-rank test (P = 0.010).

To further investigate the prognostic significance of TBX4, we first performed univariate analysis to identify factors which might affect the survival of PDAC patients. After surgical resection, the median follow-up time was 25 months. As seen in Table 2, overall survival significantly correlated with margin status, lymph node status, liver metastasis recurrence, venous invasion and TBX4 expression levels, but not with age, gender, tumor size, histopathological grading, lymphatic invasion (Table 2). Kaplan-Meier analysis revealed that low TBX4 level in PDAC tissues significantly correlated with markedly reduced overall survival (P = 0.010) (Figure 2). Additionally, we performed multivariate analysis to exclude the confounder effect. Cox proportional hazard model analysis confirmed that low TBX4 expression was an independent predictor for reduced overall survival of PDAC patients (Table 2).

### 2.4. Methylation of Promoter Region CpG Island of TBX4 in PDAC and Normal Pancreas

**Figure 3.**
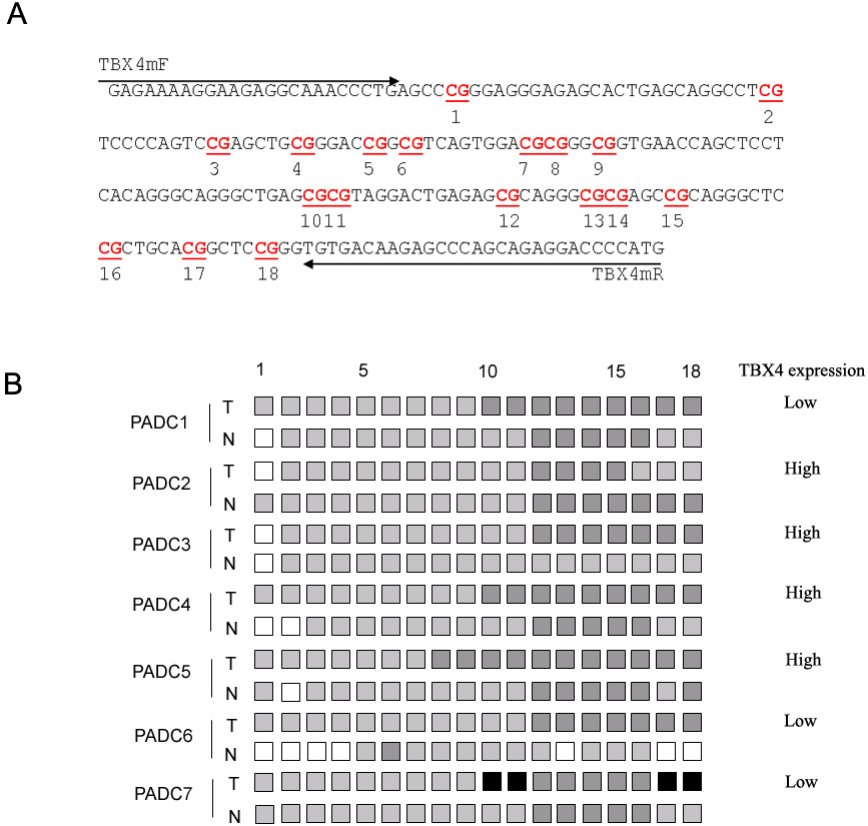
Methylation status of 18 CpG sites in the TBX4 promoter region (−1842 to −1626) were analyzed in 7 PDAC tissues (T1-7) (3 cases were defined as low level of TBX4 expression, and the other 4 cases were defined as high level of TBX4 expression) and adjacent normal pancreas tissues (N1-7) (**A**); Black, gray, and white squares represent complete methylation, partial methylation, and unmethylation status at each CpG site, respectively (**B**). Methylation status was frequently observed in both PDAC and adjacent normal pancreas tissues.

The promoter region CpG island of the TBX4 gene were analyzed in 7 PDAC tumors (including both high and low expression) and 7 normal adjacent pancreas by bisulfite sequencing. As seen in Figure 3, the CpG island regions of TBX4 promoter were hypermethylated in both PDAC and normal pancreas tissues, thus indicating that expression of TBX4 in pancreas is less likely to be regulated by DNA methylation.

### 2.5. Discussion

TBX4 belongs to the TBX gene family, which encode a group of transcription factors characterized by a highly conserved DNA-binding motif (T-box) and play a key role during organogenesis and pattern formation in both vertebrate and invertebrate embryos. Recent studies suggest that several T-box family members are implicated in the progression of multiple cancers. For example, TBX2 and TBX3 are overexpressed in several cancers including pancreatic cancer, and can contribute to cancer development and progression due to their ability to suppress cellular senescence and E-cadherin expression [10,11]. However, different from TBX2 and TBX3, in a recent study, Yu *et al*. [9] recently found that loss of TBX5 gene expression in colon cancer tissues and cell lines was associated with promoter methylation. Furthermore, TBX5 methylation was detected in 68% (71/105) of primary colon tumors, and served as a potential biomarker for the prognosis of colon cancer. In a previous proteomic study, it was shown that TBX4 was overexpressed in a proportion of patients, however, the study included only a small sample size [7]. Our data also confirm previous associations between higher TBX4 expression and better differentiated type of PDAC, and extend previous findings indicating that a low level of TBX4 expression in stage II PDACs was associated with poor overall survival and was an independent prognostic factor for patients with stage II PDACs. These findings suggested that TBX4 may play a role in the progression of pancreatic cancer.

In this study, although a nuclear pattern could be observed, the immunostaining of TBX4 protein was also located in the cytoplasm of cancer cells, which seems inconsistent with its function as a transcription factor. The cytoplasmic expression of TBX factors in cancer has been also identified in several other studies. For example, cytoplasmic overexpression of TBX2 had been identified in breast cancer tissues [12]. Qi *et al*. [7] also identified cytoplasmic TBX4 staining in pancreatic cancer tissues using another TBX4 antibody. Recently, Krcmery *et al*. characterized a functional nuclear export signal (NES) with an N-terminal DNA binding domain of all T-box proteins, by which TBX factors could shuttle between nuclear and cytoplasmic cell compartments, suggesting both nuclear and cytoplasmic roles for TBX family members [13,14]. Therefore, the above findings suggest that the cytoplasmic expression of TBX4 in pancreatic cancer could be associated with NES-directed protein shuttling; however, its functional roles in carcinogenesis remain unclear.

Among the T-box family members, TBX4 and TBX5 are highly related in their genomic organization and gene function [15,16]. Considering the possible role of TBX5 as a tumor suppressor gene in colon cancer, we speculated that loss of the Tbx4 gene might be also epigenetically controlled. However, in this study, in the CpG islands known to regulate TBX4 expression, we found that these regions were hypermethylated in both PDAC and matched normal pancreas controls through bisulfate sequencing, thus indicating that different from TBX5 expression in colon cancer, DNA methylation is less likely to regulate TBX4 expression in pancreas. The discrepancy may lie in the different patterns of gene regulation of TBX factors in different organs. Similarly, Peres *et al*. also suggested that TBX2 and TBX3, although highly homologous, have distinct roles in the transformation process of cancers where they are both overexpressed [17].

Currently available evidence indicates that TBX5 might exert its inhibitory action on cancer cells by inducing cancer cell apoptosis by targeting the extrinsic pathway, apoptotic Bax and Granzyme A signaling cascades, up-regulating the expression of a critical cell cycle inhibitor CDKN2A and an anti-metastatic gene MTSS1, or inhibiting the expression of oncogene SBCG and pro-metastatic gene MTA2 [9]. However, until now the function of TBX4 is still not well understood. Although Tbx4 might share similar molecular mechanisms with TBX5, further works need to be done to clarify its definite function in cancer development.

Pancreatic cancer represents a devastating malignancy characterized by an extremely poor prognosis, rapid progression and resistance to chemo-radiotherapy. The majority of PDAC patients with surgically resectable cancer are at stage II disease, and identifying novel tumor biomarkers may lead to better prognosis prediction and more effective treatment options for this group of patients. In this study, we have reported for the first time a significant difference in the clinical outcome among stage II patents with different TBX4 expression levels. Our data suggests that TBX4 expression level might be a useful clinical predictive factor in PDAC patients undergoing surgically resection.

This present cohort study is limited by its moderate sample size and retrospective design; therefore, further larger, multi-center and prospective clinical trials are needed to validate the clinicopathological and prognostic values of TBX4 in PDAC in the future. Moreover, the exact mechanism of TBX4 in pancreatic carcinogenesis also deserves further investigation.

## 3. Experimental Section

### 3.1. Patients

A total of consecutive 95 patients with stage II PDAC who underwent standard curative resection at Department of General Surgery, Zibo Central Hospital (Zibo, China), Qianfo mountain Hospital (Jinan, China), Shandong Provincial hospital (Jinan, China), Qilu Hospital (Jinan, China) from 1998 to 2007 were reviewed retrospectively. The median patient age at time of surgery was 64 years (range from 38–84 years). All the patients routinely received postoperative 5-fluorouracil-based chemotherapy, and Gemcitabine-based chemotherapy and radiation therapy were given when recurrence or distant metastasis occurred. Follow-up was finished in April 2009. The median follow-up was 25 months (range, 8–102 months). The overall survival (OS) time was defined as the time from operation to cancer-caused death.

Patients with PDAC were selected provided they received no preoperative chemotherapy and/or radiation, and follow-up data and archived formalin-fixed, paraffin-embedded specimens were available. Patients who died from postoperative complications were excluded. Above inclusion and exclusion criteria left 77 patients eligible for this study. The clinicopathological data of these patients are summarized in Table 1. The study was approved by the local research ethics committees at our institutions. All patients were informed with written consent obtained.

### 3.2. Immunohistochemistry

Immunohistochemical staining for TBX4 was performed on 4-μm formalin-fixed, paraflin-embedded archival tissue sections including 77 PDAC tumors and 12 normal adjacent pancreas tissues. Briefly, deparaffinized and rehydrated sections were boiled in sodium citrate buffer (pH 6.0) to perform antigen retrieval. Endogenous peroxidase activity was blocked by incubating the slides in methanol containing 3% hydrogen peroxide. Sections were incubated overnight at 4 °C with the polyclonal rabbit anti-human TBX4 antibody (Abcam, at a 1:200 dilution), and subsequently developed with an avidin-biotin-peroxidase complex method according to the manufacturer’s recommendations (Envision system, Dako). Diaminobenzidine was used as a chromogen, and hematoxylin was used as a counterstain. Non-specific rabbit IgG was used as a negative control. The expression level of TBX4 was measured as a labeling ratio between the positively staining cancer cells to the total cancer cells as described previously [18]. PDAC cases were categorized into two groups: high TBX4 expression (labeling ratio ≥25%) and low level (labeling ratio <25%).

### 3.3. Bisulfite Sequencing

Methylation of promoter region CpG island of TBX4 gene (From −1842 bp to −1626 bp, Ref mRNA: NM_018488.2) was analyzed by bisulfite sequencing as described previously [19]. Briefly, genomic DNA was extracted from 7 PDAC and 7 adjacent normal pancreas frozen tissues using the QIAamp DNA Mini Kit (Qiagen, Germany) and was modified using the Zymo EZ DNA Methylation Kit (Zymo Research) according to the manufacturer’s instructions. Modified DNA was amplified using a 20 μL mixture including 1 × HotStarTaq buffer, 2.0 mM Mg^2+^, 0.2 mM dNTP, 0.2 μM of each primer (forward 5′-GAGAAAAGGAAGAGGTAAATTTTGA-3′ and reverse 5′-CATAAAATCCTCTACTA AACTCTTATCACA-3′), 1 U HotStarTaq polymerase (Qiagen, Germany) and 1 μL template DNA using an amplification program consisting of an initial incubation for 15 min at 95 °C, followed by 11 cycles of 94 °C for 20 s, 58 °C–0.5 °C per cycle for 40 s, 72 °C for 1 min; 27 cycles of 94 °C for 20 s, 52 °C for 30 s, 72 °C for 1 min and a final 2 min incubation at 72 °C. The products of PCR were purified using SAP and Exo I, and were then sequenced on ABI 3130XL sequencing system (Applied Biosystems).

### 3.4. Statistics

The association of TBX4 protein expression with the clinicopathological characteristics was evaluated with Chi-square or Fisher’s exact test as appropriate. Overall survival in relation to TBX4 expression patterns and other clinicopathological characteristics was evaluated by Kaplan–Meier survival curve and log-rank test. Statistically significant prognostic factors identified in univariate analyses were selected to enter multivariable analyses using a Cox proportional hazards model. Statistical analyses were performed using SPSS 16.0 software for Windows. The level of statistical significance was set at p < 0.05.

## 4. Conclusions

In this study, we examined whether TBX4 expression levels are associated with survival in a cohort of 77 Stage II PDAC patients. We found that a low level of expression of TBX4 significantly correlated with poor survival compared with high TBX4 phenotype. After adjusting for clinicopathological factors, TBX4 retains an independent prognostic factor for patients with stage II PDACs in the cohort. Additionally, different from TBX5 expression in colon cancer, the expression of TBX4 in PDAC seems not to be DNA methylation-regulated.

## Figures and Tables

**Table 1.** Clinicopathological features and correlation of TBX4 expression in stage II PDACs.

**Characteristics**	**No. of cases (n = 77)**	**TBX4—high (n = 48)**	**TBX4—low (n = 29)**	**P value**
**Age (years)**				
<60	27	16	11	0.682
≥60	50	32	18	
**Gender**				
Female	32	20	12	0.980
Male	45	28	17	
**Tumor size (cm)**				
≤2.0	17	12	5	0.426
>2.0	60	36	24	
**Grade**				
1	20	14	6	0.015
2	45	31	14	
3	12	3	9	
**Margin status**				
Negative	58	35	23	0.537
Positive	19	13	6	
**Lymph node status**				
Negative	23	15	8	0.734
Positive	54	33	21	
**Liver metastasis recurrence**				
Negative	52	38	14	0.005
Positive	25	10	15	
**Lymphatic invasion**				
Negative	20	12	8	0.803
Positive	57	36	21	
**Venous invasion**				
Negative	31	21	10	0.422
Positive	46	27	19	

**Table 2.** Univariate analysis of overall survival in patients with stage II PDACs.

**Variables**	**Univariate**		**Multivariate**	

	**HR (95% CI)**	**P**	**HR (95% CI)**	**P**
**Age (years)**	1.049 (0.641–1.716)	0.846	-	-
<60 *vs*. ≥60				
**Gender**	1.534 (0.939–2.506)	0.079	-	-
Female *vs*. male				
**Tumor size (cm)**	1.437 (0.811–2.548)	0.203	-	-
≤2.0 *vs*. >2.0				
**Grade**	1.017 (0.687–1.505)	0.714	-	-
1 *vs*. 2 *vs*. 3				
**Margin status**	1.886 (1.079–3.298)	0.021	1.886 (1.049–3.390)	0.034
Negative *vs*. Positive				
**Lymph node status**	1.721 (1.037–2.857)	0.030	1.932 (1.124–3.319)	0.017
Negative *vs*. Positive				
**Liver metastasis recurrence**	1.994 (1.217–3.269)	0.004	1.455 (0.800–2.648)	0.219
Negative *vs*. Positive				
**Lymphatic invasion**	1.110 (0.656–1.879)	0.692	-	-
Negative *vs*. Positive				
**Venous invasion**	1.638 (1.015–2.644)	0.037	2.107 (1.224–3.324)	0.006
Negative *vs*. Positive				
**TBX4**	0.545 (0.337–0.881)	0.010	0.503 (0.285–0.888)	0.018
Low *vs*. High				
